# Patient–clinician discussions on lung cancer screening in the United States before and after 2021 guidelines

**DOI:** 10.1093/tbm/ibaf039

**Published:** 2025-08-13

**Authors:** Timothy J Williamson, Whitney M Brymwitt, Erin A Hirsch, McKenzie T Reese, Lisa Carter-Bawa

**Affiliations:** Department of Psychological Science, Loyola Marymount University, 1 LMU Drive, Suite 4700, Los Angeles, CA 90045, United States; Department of Psychological Science, Loyola Marymount University, 1 LMU Drive, Suite 4700, Los Angeles, CA 90045, United States; Center for Discovery & Innovation, Hackensack Meridian Health, 111 Ideation Way, Nutley, NJ 07110, United States; Georgetown Lombardi Comprehensive Cancer Center, 3800 Reservoir Rd NW, Washington, DC 20007, United States; Department of Psychological Science, Loyola Marymount University, 1 LMU Drive, Suite 4700, Los Angeles, CA 90045, United States; Center for Discovery & Innovation, Hackensack Meridian Health, 111 Ideation Way, Nutley, NJ 07110, United States; Georgetown Lombardi Comprehensive Cancer Center, 3800 Reservoir Rd NW, Washington, DC 20007, United States

**Keywords:** lung cancer screening, HINTS, USPSTF guidelines, COVID-19, patient–provider discussion

## Abstract

**Background:**

Screening for lung cancer via low-dose computed tomography of the chest can promote early detection and reduce mortality. However, since the United States Preventive Service Task Force (USPSTF) issued lung cancer screening guidelines in 2013, uptake has been low. The USPSTF revised the guidelines in 2021 to expand eligibility.

**Purpose:**

To determine whether patient–clinician discussions about lung cancer screening differs from 2017 to 2022 following 2021 revisions to the guidelines for lung cancer screening.

**Methods:**

Data were obtained from the Health Information National Trends Survey (2017, 2020, and 2022). Community-dwelling US adults (*N* = 2973) were in the eligible age range for lung cancer screening (55–80 for 2017 and 2020; 50–80 for 2022), reported current or former smoking, and had no prior history of lung cancer. The primary outcome was self-reported patient–clinician discussions about lung cancer screening within the last 12 months.

**Results:**

The weighted proportion of respondents who discussed lung cancer screening with a healthcare provider was 12.34% in 2017, 13.77% in 2020, and 9.42% in 2022. The odds of reporting screening discussions were significantly lower in 2022 than 2020 (OR = 0.58, 95% CI [0.36, 0.93]). Individuals with insurance (OR = 9.12, 95% CI [2.81, 29.96]) and those who were currently smoking (OR = 2.80, 95% CI [1.89, 4.13]) had higher odds of discussing screening.

**Conclusions:**

Patient–clinician discussions about lung cancer screening were lower in 2022 than 2020, despite revised guidelines that broadened eligibility. Research should explore strategies to increase awareness of lung cancer screening and prioritize discussions about screening among those who are uninsured and formerly smoked.

## Teaser

Fewer individuals reported having discussions about lung cancer screening with their doctor or healthcare provider in 2022 than in 2020, despite revised screening guidelines in 2021 that broadened the eligibility criteria.

Implications **Practice:** In clinical practice, healthcare providers should proactively initiate lung cancer screening discussions with screening-eligible individuals at high risk for lung cancer, especially with uninsured individuals and those who formerly smoked.

**Policy:** Healthcare policies are needed to ensure equitable access to lung cancer screening, particularly for those who are uninsured or underinsured, who had lower odds of reporting patient–clinician discussions about screening than those with insurance coverage.

**Research:** Studies should assess whether proportions of patient–clinician discussions are higher in more recent years (2023–25), following a continued return to preventive health care services after the COVID-19 pandemic and longer time for healthcare providers and the general public to be made aware of revised lung cancer screening guidelines.

Lung cancer is associated with high psychological distress and physical symptom burden and remains one of the deadliest cancers in the United States, primarily because of late-stage diagnosis [1–3]. Low-dose computed tomography (LDCT) of the chest is the best evidence-based screening tool for identifying lung cancer at an earlier, more treatable stage among high-risk individuals, which is critical for improving survival rates and facilitating supportive care to reduce morbidity [4, 5]. Specifically, the National Lung Screening Trial demonstrated the superiority of LDCT of the chest (compared to radiographic screening) in detecting earlier-stage lung cancer diagnoses and reducing lung cancer-related mortality [6, 7].

In 2013, the US Preventive Services Task Force (USPSTF) issued a Grade B recommendation for lung cancer screening with LDCT among adults aged 55 to 80 years with a 30 pack-year smoking history who were either currently smoking or quit smoking within the past 15 years [8–10]. The USPSTF recommendation was followed by the Centers for Medicare and Medicaid Services (CMS) issuing a national coverage determination for screening LDCT of the chest as a preventive health screening service in 2015, with CMS recommending screening through age 77 [11]. In 2021, in response to evolving scientific evidence [12, 13], the USPSTF and CMS revised the lung cancer screening guidelines by lowering the age and pack-year eligibility to 50 years and 20 pack-years respectively [9, 14]. This revised eligibility criteria set a promising stage for reducing observed racial inequities in lung cancer screening uptake by increasing the potential to engage underrepresented high-risk patients, such as Black and Hispanic Americans [15, 16]. The expansion in guidelines helps capture populations who have a lower pack-year history but a greater risk of lung cancer-related mortality [17].

Despite the potential early detection benefits of lung cancer screening [6, 7], uptake among those eligible has consistently been low [18–20]. Low screening uptake is multifactorial, and multilevel barriers have been identified along the lung cancer screening care continuum for eligible individuals [21, 22]. Because there are risks that must be weighed against the benefits of lung cancer screening [23], CMS requires and USPSTF recommends a shared decision-making discussion occur between a patient and an informed clinician prior to starting annual lung cancer screening [24]. These discussions are vital not only to identify those eligible for lung cancer screening but also to weigh the risks and benefits of screening [25, 26]. However, fewer than 20% of individuals potentially eligible for lung cancer screening reported having discussions with their doctor or healthcare provider about lung cancer screening in both 2012 (before the initial USPSTF guidelines) and 2014 (after the initial USPSTF guidelines) [27]. In 2020, the COVID-19 pandemic significantly disrupted many cancer screening programs [28], including lung cancer screening [29]. During this period, public and personal health efforts were understandably focused on preventing and treating COVID-19 [30], likely resulting in many eligible individuals missing critical conversations about lung cancer screening with their healthcare clinician [31]. In 2022, CMS updated its policy to allow shared decision-making discussions to be conducted by non-physician practitioners. This change enables trained auxiliary staff, such as health educators or program navigators, to lead these important conversations [32]. Therefore, the expanded screening guidelines from USPSTF in 2021 and relaxed shared decision-making requirements from CMS in 2022 may have facilitated a higher number of patient–clinician discussions about lung cancer screening, which would be an important contributor to increase overall screening participation.

The current study leveraged a large, nationally representative dataset to evaluate the proportion of people who reported ­having discussions with their healthcare provider about lung cancer screening across three time periods: 2017 (pre-COVID, under the 2013 USPSTF guidelines), 2020 (during the initial onset of COVID-19), and 2022 (under the 2021 revised USPSTF guidelines). Additionally, we examined whether survey year, race, insurance coverage, family history of cancer, and smoking status predicted lung cancer screening discussions.

## Methods

### Data source

The Health Information National Trends Survey (HINTS), conducted by the National Cancer Institute, is a nationally representative cross-sectional survey designed to gather information on the usage of cancer-related information among US adults [33]. In the current study, we used data from three recent cycles of the HINTS. HINTS 5 Cycle 1 was fielded from January to May 2017, HINTS 5 Cycle 4 was fielded from February to June 2020, and HINTS 6 was fielded from March to November 2022. All three survey cycles included a relevant question on patient–provider discussions about lung cancer screening, as well as important demographic and behavioral characteristics.

### Sample and data collection

HINTS was devised with a two-stage sampling design. In the initial stage, a stratified sample of households was selected from a database of US residential addresses. In the second stage, one adult was selected from each sampled household using the Next Birthday Method. HINTS 5 Cycles 1 and 4 used a mailed paper and pencil questionnaire which was available in both English and Spanish. HINTS Cycle 6 used two methods for data collection; participants either completed the survey via the traditional, mailed paper and pencil format or an online ­format. Additional information about the study design and sampling framework is available [34].

**Figure 1.**
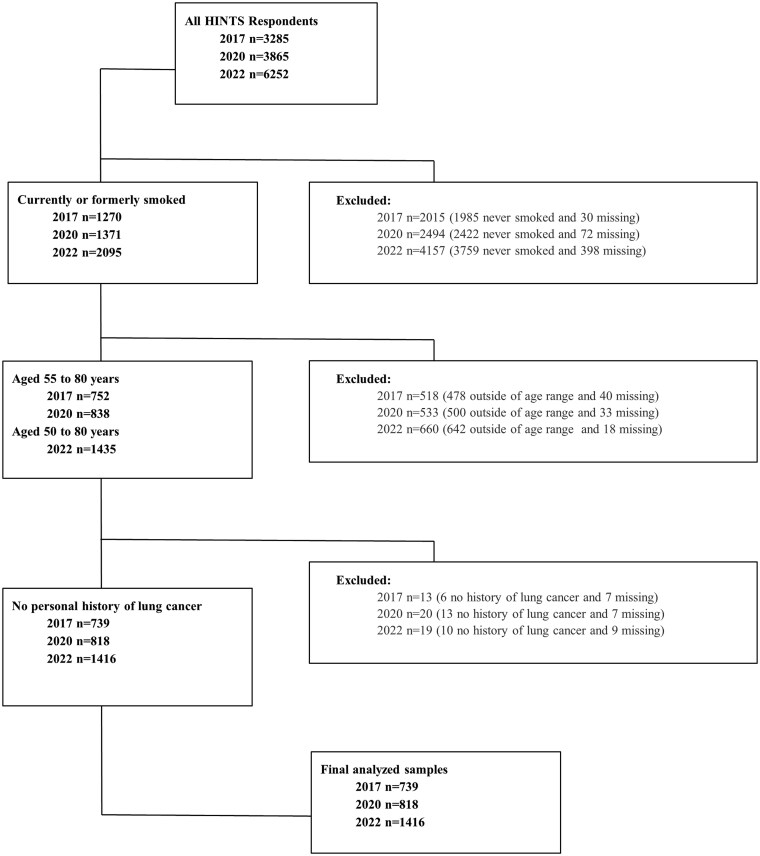
Flowchart of analyzed sample in 2017, 2020, and 2022.

The overall response rates in 2017, 2020, and 2022 were 32%, 37%, and 28%, respectively [34]. These response rates yielded a total sample size of *N* = 3285 in 2017, *N* = 3865 in 2020, and *N* = 6252 in 2022. For the present study, we analyzed data from respondents who either currently or formerly smoked, were in the eligible age range for the USPSTF lung cancer screening guidelines (ages 55–80 for 2017 and 2020; 50–80 for 2022), and with no prior history of lung cancer (see Fig. 1). Inclusion criteria were selected to approximate eligibility criteria from the USPSTF lung cancer screening guidelines [8, 9, 14], although HINTS did not measure cigarette pack-year history nor the length of time since quitting smoking. The final analyzed sample of respondents was *n* = 739 in 2017, *n* = 818 in 2020, and *n* = 1416 in 2022.

### Measures

The primary outcome was whether respondents reported having a discussion with their doctor or other healthcare professional about lung cancer screening [27]. In 2017 and 2020, the question included in the HINTS survey was “At any time in the past year, have you talked with your doctor or other health professional about having a test to check for lung cancer?”, with response options of “yes”, “no”, or “don’t know”. In 2022, this question was replaced with a new question that asks “At any time in the past year, did a doctor or other health professional talk with you about having a low-dose CT (LDCT) scan to check for lung cancer?” with the response options of “yes”, “no”, “don’t know”, or “I’ve never heard of this test”. From these questions, we generated a new outcome variable that combined responses from these questions across survey years such that responses of “no” or “I’ve not heard of this test” to either question were coded as 0, responses of “yes” were coded as 1, and responses of “don’t know” were coded as missing.

Additionally, we report on other HINTS variables including survey year (2017, 2020, and 2022), age (in years), sex (male/female), race/ethnicity (Non-Hispanic White, Non-Hispanic Black or African American, Hispanic, Non-Hispanic Asian, and Non-Hispanic Other), education (less than high school, high school, some college, and college graduate or higher), household income (less than $10 000, $20 000–$35 000, $35 000–$50 000, $50 000–$75 000, and $75 000 or more), smoking status (formerly smoked/currently smoking), family history of cancer (no/yes), and health insurance coverage (no/yes). Missing, multiple responses in error, “don’t know”, unreadable, or commission errors were recoded as missing for all analyses to enhance comparability of our findings to prior research [27, 35, 36].

### Analytic strategy

Data sets across time points were merged following the HINTS data recommendations [37]. Descriptive and inferential analyses were conducted using the jackknife replicate weights provided in the HINTS datasets, which account for sampling design, oversampling, and non-response patterns [38]. Descriptive statistics were performed among the identified subsample of respondents to produce weighted prevalence estimates for the outcome variable (discussing lung cancer screening with a healthcare provider) across each survey year and to report the distributions of sociodemographic and health status characteristic variables. A logistic regression was conducted to test whether survey year, race, insurance coverage, smoking status, or family history of cancer significantly predicted the odds of discussing lung cancer screening with a healthcare provider. To evaluate trends over time from 2017 to 2020 and 2020 to 2022, we selected 2020 as the referent group for the survey year variable. We constructed 95% confidence intervals (CIs) for the estimated odds ratios (ORs), which were used to determine statistical significance for each predictor (if the 95% CI did not include 1). Cases with missing values on the predictor(s) or outcome were excluded using listwise deletion.

## Results

Sample characteristics are presented in Table 1. Participants were significantly younger in 2022 (*M *= 62.91, *SE *= 0.37) than in 2017 (*M *= 65.35, *SE *= 0.33, *t *= 2.51, *P *< .001) and 2020 (*M *= 65.43, *SE *= 0.30, *t *= 4.91, *P *< .001), which can be explained by the inclusion of individuals aged 50–54 in the revised eligibility criteria for lung screening after the 2021 guidelines [9]. A chi-square test demonstrated no significant difference in reported lung cancer screening discussions between respondents aged 50–54 vs. 55–80 in 2022, *χ*2 = (1147) = 1.88, *P *= .173.

**Table 1.** Sample characteristics in 2017, 2020, and 2022

Variable	HINTS 5 Cycle 1 (2017), *n* = 739	HINTS 5 Cycle 4 (2020), *n* = 818	HINTS 6 (2022), *n* = 1416
	Weighted *M*(*SE*)	Weighted *M*(*SE*)	Weighted *M*(*SE*)
Age	65.35(0.33)	65.43(0.30)	62.91(0.37)
	Weighted %	Weighted %	Weighted %
Talked to doctors or health professionals about LC screening			
No	84.96	83.95	85.45
Yes	12.34	13.77	9.42
Missing	2.71	2.27	5.13
Gender			
Male	55.13	51.01	52.74
Female	40.28	44.34	46.97
Missing	4.59	4.65	0.29
Race and ethnicity			
Non-Hispanic White	71.91	74.29	72.30
Non-Hispanic Black or African American	7.17	8.26	10.39
Hispanic	7.19	5.71	8.61
Non-Hispanic Asian	0.78	1.16	1.38
Non-Hispanic other	2.80	3.11	2.73
Missing	10.15	7.48	4.59
Education (%)			
Less than high school	11.04	6.77	7.30
High school	28.61	28.32	24.03
Some college	40.45	47.27	48.17
Bachelor and post-baccalaureate	19.56	16.66	20.36
Missing	0.32	0.97	0.14
Income (%)			
Less than $10 000	21.06	16.75	14.66
$20 000–35 000	13.00	10.76	13.04
$35 000–50 000	9.66	13.40	11.30
$50 000–75 000	20.63	21.41	19.00
$75 000 or more	25.90	29.64	35.78
Missing	9.75	8.05	6.22
Smoking status (%)			
Currently smoking	68.86	71.94	69.86
Formerly smoked	31.14	28.06	30.14
Family history of cancer (%)			
No	22.08	15.48	19.95
Yes	70.99	77.35	69.81
Not sure	6.36	5.95	9.43
Missing	0.57	1.23	0.82
Insurance coverage (%)			
No	4.17	3.63	6.31
Yes	94.28	95.73	92.78
Missing	1.55	0.63	0.91

### Patient–clinician discussions about lung cancer screening from 2017 to 2022

**Figure 2.**
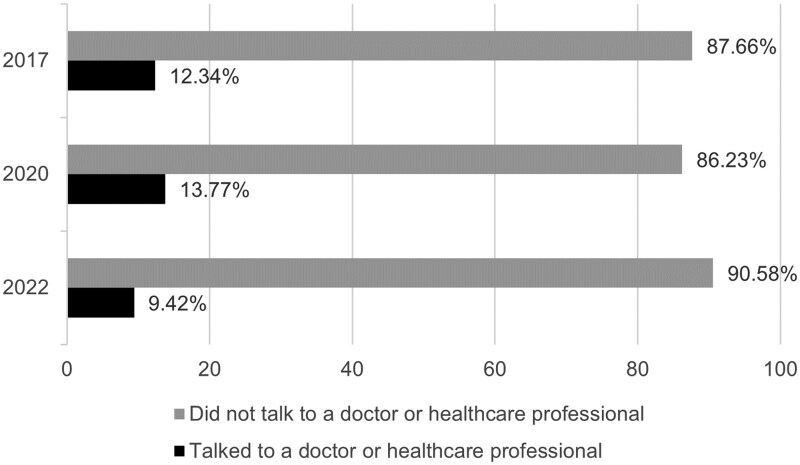
Proportions of patient–clinician discussions about lung cancer screening among HINTS respondents who were potentially eligible for lung cancer screening. Note: The weighted proportion of respondents who reported discussing lung cancer screening with a healthcare provider was 12.34% in 2017, 13.77% in 2020, and 9.42% in 2022. Participants were HINTS respondents who reported current or former smoking, were in the eligible age range for the US Preventive Services Task Force lung cancer screening guidelines (ages 55–80 for 2017 and 2020; 50–80 for 2022), and with no prior history of lung cancer. The analyzed sample of respondents was *n* = 739 in 2017, *n* = 818 in 2020, and *n* = 1416 in 2022.

The weighted proportion of respondents who reported discussing lung cancer screening with a healthcare provider was 12.34% in 2017, 13.77% in 2020, and 9.42% in 2022 (see Fig. 2). Results from the logistic regression indicate that survey year, smoking status, and insurance coverage significantly predicted patient–clinician discussions about lung cancer screening, whereas race and family history of cancer were not significant predictors (see Table 2).

**Table 2.** Logistic regression predicting patient–clinician discussions about lung cancer screening from 2017 to 2022 (n = 2403; estimated population size 96 579 736)

Variable	Adjusted OR	95% CI	Weighted population estimates of reported lung cancer screening discussions by variable category
Survey year			
2020 (referent)	1		4 556 411/30 342 234 (15.0%)
2017	0.84	[0.53, 1.34]	3 706 478/28 772 468 (12.9%)
2022	0.58*	[0.36, 0.93]	3 422 248/37 465 034 (9.1%)
Race and ethnicity			
Non-Hispanic White (referent)	1		9 053 854/76 141 017 (11.9%)
Non-Hispanic Black or African American	0.97	[0.59, 1.60]	840 828/7 435 035 (11.3%)
Hispanic	1.05	[0.45, 2.42]	1 182 641/8 780 723 (13.5%)
Non-Hispanic Asian	1.10	[0.25, 4.79]	141 750/1 206 787 (11.7%)
Non-Hispanic other	1.24	[0.47, 3.29]	466 063/3 016 174 (15.5%)
Smoking status			
Formerly smoked (referent)	1		6 601 522/70 007 071 (9.4%)
Currently smoking	2.80***	[1.89, 4.13]	5 383 614/26 572 665 (20.3%)
Family history of cancer			
No (referent)	1		2 435 626/20 448 514 (11.9%)
Yes	1.00	[0.61, 1.64]	9 249 510/76 131 222 (12.1%)
Insurance coverage			
No (referent)	1		95 065/4 841 362 (2.0%)
Yes	9.18***	[2.81, 29.96]	11 590 072/91 738 375 (12.6%)

Note: Weighted population estimates are unadjusted for covariates.

* = *P *< .05.

*** = *P *< .001.

Specifically, respondents in 2022 were more likely to discuss lung cancer screening with their doctor or healthcare provider, compared to respondents from 2020 (OR = 0.58, 95% CI [0.36, 0.93]). There was no significant difference in the proportion patient–clinician discussions about lung cancer screening in 2017 vs. 2020 (OR = 0.84, 95% CI [0.53, 1.34]) or in 2017 vs. 2022 (OR = 1.45, 95% CI [0.90, 2.33]). Respondents with insurance were more likely to discuss lung cancer screening, compared to those without insurance (OR = 9.12, 95% CI [2.81, 29.96]). Similarly, those who were currently smoking were more likely to discuss lung cancer screening, compared to those who formerly smoked (OR = 2.80, 95% CI [1.89, 4.13]). Respondents from various racial and ethnic backgrounds were not more or less likely to discuss lung cancer screening (all *P* > .665), and those with a family history of cancer were not more or less likely to discuss lung cancer screening (OR = 1.00, 95% CI [0.61, 1.64]).

## Discussion

Strikingly, the proportion of respondents who reported having discussions about lung cancer screening was significantly lower in 2022 than in 2020, despite new guidelines that broadened the eligibility criteria for screening in 2021 [9, 14]. The current findings build upon more than a decade of research dating back to the original USPSTF guideline issuance that has consistently showed low uptake of lung cancer screening among those who are eligible for screening [18–20]. Specifically, these results are similar to a prior analysis of HINTS data [27], which demonstrated a surprising finding that the proportion of patient–clinician discussions about lung cancer screening was lower in 2014 (10.4%) than in 2012 (16.7%), after the initial USPSTF guidelines were issued in 2013 [27].

The current findings may be explained by residual effects of the COVID-19 pandemic and a slow return by the general public to preventive health care services and a continued interruption to cancer screening programs during the COVID-19 pandemic in 2021 [28, 29, 39]. During the pandemic, shared decision-making conversations were rapidly moved to telephone and video-conferencing platforms [40]. Research has shown that instructions and details recalled from a telehealth visit are lower compared to details recalled from an in-person visit [41], which may contribute to the lower proportion of participants reporting a lung cancer screening discussion in 2022. The similar proportions of respondents reporting discussions of lung cancer screening in 2020 and 2017 may reflect that the HINTS 2020 survey asked people to reflect upon their interactions with healthcare providers during the prior 12 months, which included a significant period of time prior to the impact of COVID-19 on cancer screening programs. Additionally, it is possible that the lower proportion of patient–clinician discussions in 2022 reflects a need to increase health care providers’ knowledge of the USPSTF revised guidelines from 2021 [42]. There was no significant difference in reported lung cancer screening discussions between HINTS 6 (2022) respondents aged 50–54 vs. 55–80, which suggests that the lower proportion of lung cancer screening discussions in 2022 (compared to 2020) is not explained by the inclusion of respondents aged 50–54 in 2022 that reflects the broadened age eligibility criteria for lung cancer screening after the 2021 guidelines.

Our results highlight important characteristics that predict who is more likely to report having these discussions with their doctors or healthcare providers. Consistent with prior research [27], participants with insurance coverage and those who were currently smoking had greater odds of having these discussions. Given the CMS documentation requirement for shared decision-making, insurance coverage has been demonstrated consistently as a facilitator of lung cancer screening [43, 44]. This underscores an important equity gap in serving those who are uninsured or underinsured [45], as individuals without insurance coverage may lack access to preventive services and discussions about lung cancer screening [46]. Lack of insurance and access to preventive care is concerning since uninsured individuals are more likely to be diagnosed with late-stage lung cancer and have lower survival than those with private insurance and Medicare [47].

Participants who reported current smoking were more likely to report having lung cancer screening discussions with their healthcare providers. Smoking status is the most frequently known criterion for lung cancer screening among primary care providers [48]. This aligns with the focus on smoking as a primary risk factor for lung cancer and the need for targeted interventions in individuals who smoke. It may also be possible that those who formerly smoked quit more than 15 years ago, which would make them ineligible for screening and less likely to report a discussion about lung cancer screening [14]. The HINTS datasets do not contain information about years since quitting, so it is unknown how many respondents who formerly smoked are eligible for screening.

Participants’ race and ethnicity and family history of cancer were not associated significantly with reported lung cancer screening discussions. These results are consistent with some prior findings that demonstrate no racial or ethnic differences patient–clinician discussions about lung cancer or receiving a healthcare provider recommendation to screen [27, 43]. However, these results are inconsistent with findings that Black individuals are less likely to be referred by their healthcare providers for lung cancer screening (but just as likely to participate in lung cancer screening once referred) [49]. Future research is needed to explore the content of patient–clinician discussions about lung cancer screening by patients’ race to contextualize these findings further. Our findings differ from previous research demonstrating that a family history of lung cancer was a significant predictor of patient–clinician discussions of screening (from 2012 to 2014) [27]. Although family history of lung cancer is associated with higher perceived risk of being diagnosed with lung cancer [50], the nonsignificant findings in the current study may be explained by the fact that family history is neither required nor sufficient to undergo screening via LDCT [8, 9].

### Limitations and strengths

A limitation of the current study is the reliance on self-reported data, which may be subject to recall bias. Additionally, the cross-sectional nature of HINTS limits the ability to draw causal or longitudinal inferences about the factors influencing lung cancer screening discussions. Another limitation is the inability to determine whether respondents are eligible for lung cancer screening, as HINTS data does not contain information on pack-years smoked or number of years since the respondent quit smoking, potentially leading to an underreporting of lung cancer screening discussions among those who formerly smoked. Although our analysis focused on reported discussions about lung screening, it is important to note that such discussions do not indicate whether or not participants completed an LDCT. Additionally, the question about lung cancer screening changed in 2022 to specify LDCT, which may explain the smaller proportion of respondents reporting lung cancer screening discussions in 2022. Future studies should compare the 2022 responses to future HINTS datasets that use the same question specifying LDCT. Finally, there may be potential underreporting of discussions due to the stigma associated with smoking, which may affect respondents’ willingness to disclose smoking-related information.

Despite these limitations, the study has several strengths. This study leverages a large, nationally representative sample, which enhances the generalizability of the findings and offers key insights into health information in the context of patient–clinician discussions about lung cancer screening. In addition, analysis of survey cycles across different years allows for the comparison of trends across time, providing valuable insights to the rates of screening discussions before and after the USPSTF guidelines were revised. Moreover, the identification of specific predictors of lung cancer screening discussions and comparison of trends over time can guide the development of targeted interventions to address equity in lung cancer screening.

### Future directions

Future research should explore strategies to increase awareness of lung cancer screening and facilitate lung cancer screening discussions, particularly among uninsured and underinsured populations. Additionally, future research should evaluate health care provider knowledge of revised 2021 USPSTF lung cancer screening guidelines. It is important to recognize that structural and time constraints for medical appointments may hinder opportunities for shared decision-making (e.g. not enough time for questions) [51], and efforts to enhance lung cancer screening engagement should evaluate which implementation strategies (e.g. telehealth visits; centralized, decentralized, or hybrid screening programs) are best for integrating shared decision-making into their clinical workflow [32]. Interventions that address barriers to shared decision-making and enhance healthcare provider training on lung cancer screening guidelines are essential, and use of decision-aid tools that aim to increase knowledge and reduce decisional conflict may be particularly useful indicators of shared decision-making in lung cancer screening [52]. Additionally, longitudinal studies could provide more robust evidence on the impact of policy changes and other interventions on lung cancer screening uptake and adherence. Addressing these gaps will be critical to improving lung cancer outcomes and reducing health disparities. Ultimately, while the proportion of lung cancer screening discussions has not significantly increased despite broadened eligibility criteria, understanding the predictors of these discussions and the impact of external factors such as the COVID-19 pandemic can inform future efforts to enhance lung cancer screening rates and promote early detection.

## Welfare of Animals

This article does not contain any studies with animals performed by any of the authors.

## Transparency Statement

*Study Registration:* This study was not formally registered.

*Analytic Plan Pre-Registration:* The analysis plan was not formally pre-registered.

*Analytic Code Availability:* The data is publicly available at https://hints.cancer.gov/data/. Analytic code used to conduct the analyses presented in this study are not available in a public archive but may be requested by emailing the corresponding author (timothy.williamson@lmu.edu).

*Materials Availability:* All materials used to conduct the study are available in a public archive at https://hints.cancer.gov/data/.
